# Digitale Plattformen zur Vermittlung informell Helfender für die häusliche Pflege: Welchen Nutzen sehen Anbieter?

**DOI:** 10.1007/s00391-023-02252-6

**Published:** 2023-11-08

**Authors:** Tobias Wörle, Jochen Geiselhart, Christian Haushammer, Dominik Bernhard

**Affiliations:** Bayerisches Zentrum Pflege Digital, Hochschule Kempten, Albert-Einstein-Str. 6, 87437 Kempten – St. Mang, Deutschland

**Keywords:** Häusliche Pflege, Nutzen, Befragung, Freiwilliges Engagement, Qualitätsverbesserung, Home care, Benefits, Survey, Volunteerism, Quality improvement

## Abstract

**Hintergrund:**

Über den tatsächlichen Nutzen digitaler Plattformangebote, die engagementbereite Personen an Haushalte mit Pflegeaufgaben vermitteln, ist bisher wenig bekannt.

**Ziel:**

Der Beitrag stellt erste Ergebnisse einer Vermittlungsanbieterbefragung als Teil einer mehrstufig angelegten Interviewstudie vor. Im Zentrum steht zunächst die Frage, welche Nutzenaspekte Anbieter ihren Angeboten im Hinblick auf die eigene Organisation, die Nutzer:innen sowie auf gesellschaftlicher oder sozialräumlicher Ebene zuschreiben.

**Material und Methoden:**

Für leitfadengestützte Interviews wurden Vermittlungsanbieter ausgewählt. Zwei davon nutzen eine Matching-Plattform, zwei nicht. Die Daten wurden über inhaltsanalytische Verfahren ausgewertet.

**Ergebnisse:**

Effizienzvorteile, Qualitätsgewinne, aber auch neue Möglichkeiten zur Erschließung weiterer Helferklientele sind aus Anbietersicht wichtige Nutzenaspekte digitaler Matching-Plattformen für die eigene Organisation. Um Vertrauen und Akzeptanz aufseiten der Nutzenden zu fördern, erscheinen hybrid gestaltete Vermittlungsmodelle besonders vorteilhaft. Sie ermöglichen neben der Nutzung digitaler Technologien zugleich persönliche Begleitung im Aufbau von Hilfebeziehungen. Die Befunde legen zudem nahe, dass gemeinwohlorientierte, qualitätsgesicherte Vermittlungsplattformen auch auf sozialräumlicher Ebene Nutzenvorteile entfalten können.

**Diskussion und Fazit:**

Neben Nutzenaspekten in Bezug auf die eigene Organisation und auf Nutzer:innen konnten auch nutzenstiftende Potenziale in Bezug auf lokale Sorgenetzwerke rekonstruiert werden.

## Abstract (en)

**Background:**

Little is known so far about the actual benefits of digital platforms that mediate volunteer helpers with households facing care responsibilities.

**Objective:**

This article presents the initial results of a survey conducted among placement agencies as part of a multistage organized interview study. The key question is what benefits do agencies ascribe to the services in their own organization for the users and on a societal or sociospatial level.

**Material and methods:**

For the purpose of conducting explorative guideline-based interviews, four placement agencies were selected. Of these, two use a matching platform and two do not. The data were analyzed using content analysis methods.

**Results:**

The providers perceive advantages in efficiency, improvements in quality but also new possibilities for harnessing new helper clientele as important beneficial aspects of using digital placement platforms for their own organization. In terms of nurturing users’ trust and acceptance, hybrid matching models appear to be particularly advantageous by deploying digital technologies and also enabling personal support in developing helping relationships. The findings also suggest that public welfare-oriented, quality-assured matching platforms can unfurl additional benefits at sociospatial and societal levels.

**Conclusion:**

Besides beneficial aspects for organizations and users it was also possible to reconstruct potential benefits in relation to local care networks.

Vor dem Hintergrund des demografischen Wandels und des Pflegefachkräftemangels wird den Unterstützungsleistungen von freiwillig engagierten Menschen bei der Versorgung pflegebedürftiger älterer Menschen eine zunehmende Bedeutung zugesprochen [2]. Um diesen Versorgungsengpässen auch mithilfe freiwillig Engagierter besser begegnen zu können, wird auf digitale Lösungen wie digitale Plattformen verwiesen, auch wenn die Potenziale und Auswirkungen des Einsatzes digitaler Plattformen im Kontext von Gesundheit, Pflege und Sorge kontrovers diskutiert werden [14]. In digitalen Plattformangeboten, die engagementbereite Einzelpersonen^1^ an hilfesuchende Haushalte mit Pflegeaufgaben vermitteln, lassen sich aber auch Potenziale vermuten. So sollen über digitale Vermittlungsplattformen brachliegende Engagementpotenziale aktiviert werden und die über die Plattform vermittelten, freiwilligen Helfer:innen professionelle Pflege- und Betreuungsleistungen ergänzen, indem sie pflegende Angehörige durch haushaltsnahe Dienstleistungen im Alltag entlasten [8, 13, 14].

## Forschungsstand

Nutzung und Nutzen von Vermittlungsplattformen im Kontext des freiwilligen Engagements für hilfesuchende Haushalte mit Pflegeaufgaben wurden noch kaum empirisch beforscht. In einigen wenigen Beiträgen zu Engagementplattformen [1] oder zu Gig-Work-Plattformen in der Pflege [7, 13, 14] werden Nutzenaspekte wie höhere Transaktionszahlen und die einfache Verwaltung, Akquise oder Evaluation von Klient:innen als relevanter Nutzen für Anbietende erwähnt. Der Aufbau von Vertrauen zwischen den Matching-Partner:innen gilt als besonders erfolgskritisch, ebenso die Herstellung von Vertrauen dem Anbieter gegenüber – z. B., ob Nutzende von der Verlässlichkeit und Kontinuität des Angebots überzeugt sind [14] oder digitale Vermittlungsangebote überhaupt akzeptieren [13]. Auch Maßnahmen zur Qualitätssicherung gelten als erfolgskritisch [7]. Für Nutzer:innen wird neben den geringeren Transaktionskosten auf die Personalisierung des Angebots, die Individualisierung von Leistungen und Möglichkeiten zur Überbrückung räumlicher Distanz verwiesen. Auch könnten mit digital unterstützten Plattformen mehr Transparenz (z. B. durch Bewertungssysteme), Flexibilität und Komfort entstehen [14]. Es besteht jedoch Konsens, dass nicht von einer allgemeingültigen Plattformlogik auszugehen ist, sondern „Plattformen … stets in ihrem spezifischen Nutzungskontext“ zu betrachten seien [3, S. 119].

## Zielsetzung

In zunächst explorativer Absicht erkundet das Projekt „NuVe – Nutzen von Vermittlungsplattformen für informell Helfende in der häuslichen Pflege“ (Laufzeit: 2022–2023) in einer mehrstufig angelegten qualitativen Interviewstudie, welchen Nutzen Anbieter, helfende sowie hilfesuchende Nutzer:innen entsprechenden Plattformangeboten zuschreiben. Im ersten Schritt wurden Anbieter von Vermittlungsangeboten mit und ohne Matching-unterstützender Plattform befragt. Nachfolgend werden erste Ergebnisse dieser Anbieterbefragung vorgestellt. Die darin erfassten Nutzenperspektiven werden im Hinblick auf drei Dimensionen (Organisation, Individuum, Gesellschaft bzw. Sozialraum) diskutiert.

## Theoretische Grundlagen

Nutzenzuschreibungen an digitale Technologien erfolgen ausgehend von subjektiven, akteursgebundenen Perspektiven und sind variabel. Insofern kann nicht von „dem Nutzen“ gesprochen werden [6, 16]. Vielmehr geht das Projekt NuVe von einem multidimensionalen, relativen Nutzenverständnis aus. Das „Nutzenmodell zur Anwendung von Assistenztechnologien für pflegebedürftige Menschen“ (NAAM) [12] integriert unterschiedliche Ansätze zur Evaluation digitaler Technologien. Dem Modell wurde entlehnt, Nutzen als mehrdimensionales Konstrukt auf verschiedenen Ebenen zu betrachten, etwa einer individuellen, auf die Einzelperson bezogenen Ebene, einer struktur- und prozessbezogenen Ebene (z. B. im Versorgungsprozess für Pflegeempfangende) oder Bedingungen, unter denen eine Technologie genutzt wird. Da für Matching-Plattformen, die Hilfe vor Ort vermitteln, auch auf anderen Ebenen wie der Gesellschaft Nutzenpotenziale vermutet werden können [1], wurde der Fokus um sozialräumliche und gesellschaftliche Ebenen erweitert.

Digitale Matching-Plattformen werden verstanden als transaktionszentrierte digitale Angebote, die kommunikative und koordinierende Schnittstellen zwischen verschiedenen Akteuren schaffen [1, 7]. Als hybride Plattformen werden gemeinhin Vermittlungsangebote, die nicht allein die Herstellung einer Transaktion, sondern auch das Wohlergehen der vermittelten Personen im Blick haben, bezeichnet [10]. Von hybriden (Vermittlungs‑)Angeboten ist die Rede, wenn darin unterstützende digitale Technologien eng mit nichtdigitalen Elementen wie etwa personengestützten Beratungs- oder Begleitangeboten durch angestelltes Fachpersonal für die Klientel verschränkt sind.

## Methoden

Um Nutzenperspektiven von Anbietern aus deren subjektiver Sicht erfassen zu können, wurden offene Leitfadeninterviews genutzt [15]. Neben zunächst freien Erzählungen der Anbieter zu Nutzen, Nutzung und Ablauf des Vermittlungsprozesses wurde im Rahmen erzählgenerierender Nachfragen auch auf mögliche Nutzenaspekte, die vorab aus der Literaturdurchsicht gewonnenen werden konnten, zurückgegriffen. Die Daten wurden anhand qualitativer Inhaltsanalysen nach Kuckartz [11] ausgewertet. Schlüsselpassagen in den Transkripten wurden vertiefend im Zuge hermeneutischer Verfahren analysiert [11].

Die Fallauswahl folgte dem Prinzip des „theoretical sampling“ der Grounded Theory-Methodologie [4]. Vorab eingrenzende Kriterien zur Auswahl von Anbietern waren ein in Deutschland verorteter Standort sowie die Vermittlung engagementbereiter Privatpersonen als Helfende vorwiegend im Kontext altersassoziierter Pflege und Sorge im Rahmen von Alltagsunterstützung. Als erster Fall wurde ein etablierter Anbieter (A1) ausgewählt; dieser nutzt eine digitale Vermittlungsplattform. Nach globalen Auswertungen des ersten Interviews mit dem Geschäftsführer dieses Anbieters wurde als minimaler Kontrast der Vorsitzende einer Genossenschaft (A2), dessen Betriebs- und Vermittlungsmodell dem von A1 ähnelt und der dieselbe digitale Plattform zur Hilfevermittlung nutzt, befragt. Die Plattformen der Anbieter A1 und A2 sind nicht miteinander vernetzt, sondern voneinander unabhängige Lizenzen des gleichen Softwareprodukts. Als maximale Kontraste wurden die Koordinatorin eines städtischen Angebots (A3) bzw. der Vorstand eines Vereins (A4), deren Betriebs- und Vermittlungsmodelle dem von A1 gegenüber stark abweichen, befragt (Tab. 1). Alle Anbieter operieren mit ihren Vermittlungsangeboten im lokalen Kontext und vermitteln Hilfe im Rahmen ihrer (Groß‑)Stadtgrenzen. Die Hilfsbeziehungen selbst finden im kleinräumigen Radius von 1–5 km statt.

**Table 1:** 

	A1	A2	A3	A4
Organisationsform	Eingetragener Verein	Eingetragene Genossenschaft	Kommunales Angebot	Eingetragener Verein
Reichweite	Metropole (> 1 Mio. EW)	Regionalzentrum (250.000 EW)	Oberzentrum (75.000 EW)	Marktgemeinde (9000 EW)
Auftragsvolumen	Ca. 800 Aufträge im Monat	Ca. 200 Aufträge im Monat	Ca. 15–30 aktive Hilfebeziehungen im Monat, mit unregelmäßigen Aufträgen	Ca. 100 Aufträge im Jahr
Digitale Plattform	Ja (mit Mobil-App und Webclient für Helfende)		Nein (vorwiegend über Telefon und Kontaktlisten)	

*EW* Einwohner

Zum besseren Verständnis soll exemplarisch die Funktionsweise der untersuchten digitalen Plattform von A1 und A2 am Beispiel des Anbieters A1 erläutert werden. Auf der digitalen Plattform können Hilfsangebote und Hilfsgesuche von engagementbereiten und hilfesuchenden Menschen erfasst werden. Helfende und Hilfesuchende können per Telefon, E‑Mail oder über eine Geschäftsstelle Kontakt aufnehmen. Anschließend werden Profile, die kurze Personenbeschreibungen, angebotene bzw. angefragte Hilfstätigkeiten, zeitliche Verfügbarkeit und Wohnort enthalten, erstellt. Diese Profile werden in der Plattform hinterlegt. Die Vermittlungsanbieter können dann mithilfe eines Plattformalgorithmus automatisch die Profildaten von Helfenden und Hilfesuchenden abgleichen und einen sog. Match (Vermittlungstreffer, basierend auf den Profildaten) erzeugen. Die Helfenden erhalten daraufhin eine automatisierte Anfrage von der digitalen Plattform per App, E‑Mail, Webclient oder in Ausnahmefällen auch per Anruf des Vermittlungsanbieters. Diese Anfragen können von den Helfenden zu- oder abgesagt werden. Wird eine Hilfsanfrage angenommen, erhalten die Helfenden die Kontaktdaten der Hilfesuchenden und können ein Kennenlernen bzw. ‚Probehelfen‘ vereinbaren. Zusätzlich haben die Helfenden auch die Möglichkeit, auf der Plattform selbstständig nach Hilfsgesuchen zu recherchieren. Die Anbieter ohne digitale Plattform verwenden eine Helferkontaktliste und vermitteln, indem sie Hilfsgesuche manuell mit den Daten der Helfenden abgleichen.

## Ergebnisse

### Organisations- und klientelbezogene Nutzenperspektiven

Vorteile für die eigene Organisation sehen die Anbieter mit plattformunterstützten Vermittlungsmodellen zum einen in Effizienzgewinnen (z. B. einfache Abstimmung im Fallmanagement, geringerer Personalbedarf, zeitsparendes Matching, Möglichkeiten zur Verwaltung größerer Helfenden-Pools). Zum anderen werden Qualitätsgewinne geschildert (z. B. hohe Passgenauigkeit im Matching durch eindeutig filternde Profilinformationen, etwa zu Art und Zeitrahmen benötigter Unterstützung). Für die Erschließung neuer Typen von Engagierten wird die „neue Unverbindlichkeit“ (A2) digitaler Plattformen nicht als Problem, sondern als Vorteil gesehen („Ich lege mir mal ein Profil an. … Kannst ja alles wieder löschen. Dann hast du … dadurch einen neuen Zugang gewonnen. Neue Zielgruppen erschlossen.“ A2).

Digitale Plattformen ermöglichen die Verwaltung größerer Helfendenzahlen, was Hilfesuchenden größere Diversität in den (auch zeitlich) verfügbaren Helfendenkompetenzen biete (A1, A2). Ein zeitnaher Matching-Erfolg wird wahrscheinlicher. Das fördert auch zeitliche Flexibilität. Komfortfördernde Aspekte für ihre Klientel sehen A1 und A2 zudem in der Personalisierung des Angebots, der Bedienungsfreundlichkeit von App und Webclient, aber auch in einfachen Abrechnungsformen für erbrachte Leistungen.

Auch Anbieter ohne Matching-Software (A3, A4) erkennen in der digitalen Unterstützung durch Plattformen durchaus Vorteile für die eigene Organisation (i. e. Effizienzgewinne, Vorteile für die Qualitätssicherung, neue Skalierungsmöglichkeiten, geringe Fehleranfälligkeit durch Datenbanklösungen, schnellere Kommunikationswege und Matching-Erfolge bei geringeren Transaktionskosten). Die bisherige Nichtnutzung begründen A3 und A4 v. a. mit organisationsspezifischen Rahmenbedingungen (bei A4 fehlende Ressourcen und Kompetenzen und hoher Altersdurchschnitt im Verein und unter Nutzer:innen; bei A3 auch Hürden in der Kommunalverwaltung). Für die Zukunft finden A3 und A4 hybrid gestaltete Angebotsmodelle, die trotz digitaler Plattformunterstützung engen persönlichen Kontakt mit der Klientel erlauben, attraktiv.

### Nutzenperspektiven in Bezug auf nichtdigitale Vermittlungsaspekte

A1 und A2 ist trotz Matching-Algorithmen die persönliche Begleitung und Beratung ihrer Klientel wichtig, weil sie das Sicherheitsempfinden, die Qualitätsüberzeugungen und die Akzeptanz auf Klient:innenseite fördern. Da auch persönliche Betreuungsangebote bestehen (bei A1 u. a. begleiteter Erstkontakt) bekämen Nutzende von den digitalen Prozessen im Hintergrund oft gar nichts mit (A1, A2).

Eine besondere Rolle spielt bei den Anbietern die Qualifizierung der Helfenden durch Alltagshilfeschulungen. Diese Schulungen dienen als Vorbereitung auf die Unterstützung älterer Menschen und den Umgang mit herausforderndem Verhalten, z. B. infolge einer demenziellen Erkrankung. Neben der Überprüfung der polizeilichen Führungszeugnisse werden diese Schulungen als zusätzliche Sicherheitsmaßnahme genutzt, um ein Gespür für die zukünftigen Helfenden zu bekommen und diese einschätzen zu können. Diese Maßnahmen werden einerseits in Bezug zur Qualität des Angebotes gesetzt, andererseits auch im Kontext der Vertrauensbildung für die unterstützten älteren Menschen diskutiert. Nicht zuletzt legt besonders A1 starken Wert auf ein vertrauenswürdiges Auftreten der Organisation und betrachtet die Helfenden als „Visitenkarte nach außen“ (A1).

Aus Gründen der Sicherheit und Angebotsqualität wird etwa bei A2 vor dem Onboarding auf der Plattform stets zumindest telefonischer Kontakt hergestellt, um die Seriosität der engagementbereiten Person einzuschätzen. Gerade bei schwer vermittelbaren Personen spielen persönliches Kontextwissen und das Gespür des Personals für die individuellen Bedarfslagen eine wichtige Vermittlungsgrundlage jenseits des Matching-Algorithmus („Und dann hast Du ja noch ein paar im Hinterkopf, … die du persönlich kennst.“ A2).

In den Erzählungen von A3 und A4 fallen starke Verpflichtungsmotive dem örtlichen Gemeinwesen gegenüber auf. Auch nehmen beide ein hohes Maß an Verbindlichkeit, Erreichbarkeit und Sensibilität für die individuellen Bedarfe ihrer Klientel für sich in Anspruch. In Verbindung mit der engen persönlichen Begleitung (beispielsweise, indem stets eine dritte Person in die Hilfebeziehung integriert sei; A3) führe das auf Klient:innenseite zur Wahrnehmung großer Sicherheit und Zuverlässigkeit (A3, A4). Die einerseits begrenzte Reichweite erleichtert aus Sicht von A3 und A4 andererseits den persönlichen Kontakt zur Klientel in ihrem lebensweltlichen Kontext. Infolgedessen erlebten auch die Helfenden ihr Engagement als besonders effektiv und motivierend (A3).

Digitale Barrieren werden von A3 und A4 bewusst vermieden. Neben Telefon und Print-Informationen spielen Begegnungsorte wie Gemeindecafé, Bürgertreff oder Präsenz in Sportvereinen eine wichtige Rolle. Die Identifikation mit dem Ort und geteilte sozialräumliche Bezüge wirkten auf Helfende und Hilfesuchende vertrauensbildend und motivierend (A4). Die Gewissheit, dass hilfsbereite Personen meist derselben Gemeinde angehören oder gar „vom Sehen“ schon bekannt sind, schaffe zusätzliches Vertrauen. Das nähmen ältere Hilfesuchende klar als Nutzenvorteil wahr (A4). Der Aufbau interpersonalen Vertrauens [17] zwischen Matching-Partner:innen wird so erleichtert. Nicht zuletzt dadurch hebe man sich von Online-Anbietern ab (A4).

### Sozialraum- und gesellschaftsbezogene Nutzenperspektiven

Die Nutzenargumentationen der befragten Anbieter umfassen auch Aspekte auf der sozialräumlichen und gesellschaftlichen Dimension, die nicht allein an der Nutzung oder Nichtnutzung der digitalen Matching-Software festzumachen sind. Dabei zeigen sich Zusammenhänge mit Nutzenaspekten auf der individuell-personenbezogenen Dimension.

Alle Anbieter verstehen ihr Angebot nicht nur als Beitrag zur Bewältigung des gesamtgesellschaftlichen Problems des Versorgungsnotstands (insbesondere A1), sondern mehr oder weniger auch zur Förderung von Solidarität und Teilhabe durch die Unterstützungsbeziehungen vor Ort. Diese manifestieren sich aufgrund der stark lokal erbrachten Hilfs- und Unterstützungsleistungen und zeigen sich zunächst in den jeweiligen Quartieren. Dies v. a. in jenen Fällen, deren Klientel lokal enger eingegrenzt ist und die in den sozialräumlichen Bezügen vor Ort besonders verhaftet sind (A3 und A4, aber auch A2). A2 betrachtet die Vermittlung in eine Hilfebeziehung als „[g]leichzeitig … eine Struktur und … Vorfühlen von Arbeitsmarkt. … Regeln eben im Leben“ (A2). Darüber eröffnen sich auch für Menschen mit psychischen Erkrankungen oder eine „spezielle Klientel“ (A2), Menschen, die nicht ohne weiteres auf dem Arbeitsmarkt Fuß fassen, neue Jobperspektiven und Teilhabechancen. A2 sieht es auf der individuellen Ebene zunächst als sozialen Beitrag an, mit dem Vermittlungsangebot Zuverdienstmöglichkeiten für Helfende zu schaffen („3000 Euro ist auf Hartz IV echt viel Geld“), verspricht sich aber auch positive Folgeeffekte im Sozialraum. Denn es besteht eine Kooperation mit dem sozialpsychiatrischen Dienst, dessen Klient*innen aufgrund ihrer psychischen Auffälligkeiten oft keine Hilfe bekommen. Diese vermeintlich schwer zu vermittelnden Klienten bekommen bei A2 dann oft Unterstützung durch besagte Helfende: „[D]a …, kriegen wir da auch … ein gutes Match hin. Das mögen die Sozialarbeiterinnen …, wenn sie endlich einen Klienten … versorgt kriegen.“ An diesem Beispiel wird auch deutlich, dass es häufig nicht allein um die Herstellung eines instrumentellen Unterstützungsverhältnisses geht, sondern – aus Sicht von A2 – um „echte Hilfebeziehungen“, im Idealfall um „Freundschaft“ (A2) zwischen Helfenden und Hilfesuchenden im Sozialraum. Denn davon ausgehend könne sich Vertrauen aufbauen, und „[m]it dem Vertrauen … die Verantwortung“ (A2), was sozialen Zusammenhalt und Solidarität vor Ort fördere. Letztlich geht es um den Aufbau von Grundlagen für neues Systemvertrauen [9].

Angesichts der großen Nähe zu Hilfesuchenden in ihrer Lebenswelt zielen auch A3 und A4 auf präventive und stützende Effekte im Sozialsystem ab. Würden sich in Gesprächen Probleme oder ungesehene Bedarfe zeigen, werde dem nachgegangen und das Hilfe- und Versorgungssystem aktiviert (A4). Aus Sicht von A3 und A4 ist dies ein erheblicher Nutzenvorteil. A4 versteht sein Vermittlungsangebot auch explizit als Ergänzung der kommunalen Daseinsvorsorge.

## Diskussion

### Dimension eigene Organisation

Von allen Anbietern werden neben Effizienz- und Qualitätsvorteilen die Vertrauenswürdigkeit, Akzeptanz und fachliche Qualität des Angebots [13] als nutzenrelevant angesehen und aktiv bearbeitet. Darüber hinaus sehen Otto et al. [14], „menschliche Qualität sowie berechenbare und entlastende Kontinuität“ bzw. Zuverlässigkeit sicherzustellen, als Schlüsselbedingung für den Erfolg. In dieser Hinsicht machten die Ergebnisse teils sehr klar definierte Kriterien und Maßnahmen für Sicherheit und Angebotsqualität deutlich (z. B. Prüfung von polizeilichen Führungszeugnissen der Helfenden, Supervisionsangebote für beide Parteien der Hilfsbeziehung oder Helfer:innenschulungsangebote zur Vorbereitung auf die Alltagshilfen für ältere Menschen). Aber auch die persönliche Begleitung nimmt für die softwareunterstützten, stärker professionalisierten Anbieter einen hohen Stellenwert für den Erfolg ein. Vertrauen, Akzeptanz und den Eindruck von Zuverlässigkeit fördern Anbieter ohne Matching-Software hingegen insbesondere durch informelle Kontakte zur Klientel, eine starke Verankerung in den umgebenden sozialräumlichen Strukturen und die starke Identifikation mit dem Ort ihres Wirkens.

Auf unterschiedliche Weise scheint zudem allen befragten Anbietern eine mehr oder weniger starke Individualisierung und Personalisierung ihres Angebots zu gelingen. Auch dies stützt Einschätzungen zu nutzenrelevanten Erfolgskriterien aus anderen Anwendungskontexten [7, 13].

### Individuell-persönliche Dimension

Auf der individuell-persönlichen Dimension lassen sich aus den Befunden zu Anbietern mit Matching-Software Entlastungspotenziale für Hilfesuchende und Helfende ableiten, die ebenfalls Befunde aus anderen Studien stützen (i. e. Effizienz‑, Qualitäts- oder Komfortvorteile, aber auch größere Vielfalt an Kompetenzen und zeitliche Verfügbarkeit durch größere Pools an Helfenden).

Hybrid gestaltete Vermittlungsmodelle scheinen besondere Qualitäts- und Vertrauensvorteile zu bieten. Persönliche Begleitung und Überprüfungen stellen für Hilfesuchende die Zuverlässigkeit und fachliche Qualität sicher – womit nicht zuletzt auch psychologische Entlastungseffekte unterstellt werden dürfen. Die Verschränkung der digitalen Kontaktkanäle auf der Plattform mit herkömmlichen Kontaktkanälen, wie etwa Telefon oder persönlichen Gesprächen, erlaubt ein vereinfachtes Vermittlungsmodell, ohne digitale Barrieren zu schaffen.

### Sozialräumliche und gesellschaftliche Dimension

Die Möglichkeiten zur Skalierung in der Fläche und zur Steigerung der Transaktionszahlen erscheinen auch für die digital unterstützten Vermittlungsanbieter begrenzt und verschärfen Herausforderungen in der Vertrauensbildung. Helfende bleiben mit ihren Unterstützungsleistungen mehr oder weniger ortsgebunden. Hier kommt offensichtlich das Spannungsfeld zwischen den neuen Möglichkeiten räumlicher Entgrenzung durch Digitalisierung auf der einen Seite und dem Trend zur (Re‑)Regionalisierung auf der anderen Seite zum Tragen [14].

Die Steigerung ihres gesellschaftlichen Nutzens könnte künftig ein entscheidendes Erfolgskriterium für Vermittlungsplattformen im Kontext Pflege und Sorge sein [13, 14]. Für die untersuchten Anbieter scheint dies bereits Teil ihrer Mission zu sein. Hier soll nochmals auf die Ergebnisse zu solidaritäts- und teilhabefördernden Nutzenargumenten zurückverwiesen werden. Denn vor diesem Hintergrund erscheinen in der Tat „gemeinwirtschaftlich und … sozialräumlich orientierte Plattformen, die sich konsequent an Qualität und am Nutzen der Beteiligten orientieren“ [14] als vielversprechende Alternative zu Gig-Work-Plattformen mit schlankeren Geschäftsmodellen [10].

Die präsentierten Kriterien und Dimensionen gilt es, im Projekt *NuVe *mit Nutzenperspektiven von helfenden und hilfesuchenden Nutzer:innen der Vermittlungsangebote in Beziehung zu setzen; diese werden im nächsten Schritt erhoben.

## Fazit für die Praxis

- Effizienzvorteile, Qualitätsgewinne und neue Möglichkeiten zur Erschließung neuer Helfer:innen sind aus Anbietersicht wichtige Nutzenaspekte digitaler Matching-Modelle für die eigene Organisation.
- Hybrid gestaltete Vermittlungsmodelle fördern Vertrauen und Akzeptanz der Nutzenden.
- Gemeinwohlorientierten, qualitätsgesicherten Vermittlungsplattformen können auch auf gesellschaftlicher und sozialräumlicher Ebene Nutzenvorteile unterstellt werden. In dieser Hinsicht könnten sie lokale Sorgenetzwerke potenziell nutzenstiftend ergänzen.
- Eine weitere wissenschaftliche Fundierung der Nutzenpotenziale digitaler Matching-Plattformen im Kontext häuslicher Pflege, lokaler Sorgenetzwerke und freiwilligen Engagements ist wünschenswert.

## Footnotes

1 Freiwilliges Engagement wird im weiteren Text als Oberbegriff für alle Formen ehrenamtlichen oder freiwilligen Engagements verwendet [5].
